# Tanshinone for polycystic ovary syndrome: A protocol of systematic review and meta-analysis

**DOI:** 10.1097/MD.0000000000024287

**Published:** 2021-01-22

**Authors:** Yijiao Yang, Yue Xia, Xia Peng, Jiani Xie, Honglin Liu, Xiaorong Ni

**Affiliations:** Gynecology Department, Shanghai Traditional Chinese Medicine Hospital, Shanghai, China.

**Keywords:** polycystic ovary syndrome, randomized controlled trials, systematic review, tanshinone

## Abstract

**Backgrounds:**

Polycystic ovary syndrome (PCOS) constitutes an endocrine and metabolic disorder characterized by hyperandrogenemia, ovulation disorders, and polycystic ovary. Existing therapy is low efficacy and has significant side effects. In traditional Chinese medicine, tanshinone was used for PCOS women. Here, we will investigate the safety, as well as the efficacy of tanshinone in treating polycystic ovary syndrome.

**Methods:**

Two researchers will independently research eligible randomized controlled trials in 6 repositories: PubMed, CINAHL, Web of Science, EMBASE, China National Knowledge Infrastructure (CNKI), as well as Cochrane Library, from their onset to present. The languages will constitute either English or Chinese, and we will carry out article selection, data mining, and conduct an evaluation of the risk of bias by the Cochrane tool of risk of bias. All analyses will be conducted by using the Cochrane Review Manager software (RevMan 5.3).

**Results and conclusion:**

This study will provide the latest research evidence on the efficacy, as well as safety of tanshinone for PCOS patients.

**Registration number:**

INPLASY2020100017

## 1 Introduction

PCOS is a common, complex, and diverse endocrine and metabolic disorder characterized by hyperandrogenemia, ovulation disorders, and polycystic ovary.^[1]^ It affected about 8% to 13% of reproductive age women around the globe according to 3 main diagnostic criteria.^[2]^ To date, it is the leading cause of anovulatory infertility, about 50% to 70% anovulatory infertility cases.^[3]^ PCOS poses a significant risk for metabolic diseases, type 2 diabetes, insulin resistance (IR), cardiovascular disease, endometrial cancer, and pregnancy complications.^[4–7]^ Therefore, PCOS not only has an impact on a person's health, as well as quality of life but also on the social-economic burden. So early diagnosis and treatments are essential.^[8]^

The etiology of PCOS is still unclear, mainly genetic factors, lifestyle, etc.^[9]^ So far, the current diagnosis remains controversial, which partly contributed to the limitation of the available therapeutic options. In general, the individual's symptoms and the patient's wishes to conceive determine actual treatment decisions. Ovulation disorder is one of the leading clinical manifestations of PCOS. Consistently, clomiphene and letrozole are listed as traditional ovulation-inducing agents. In recent years, it is mostly thought that IR is the leading cause of ovulation disorder in PCOS women.^[10]^ However, on the one hand, clomiphene and letrozole have considerable side effects. On the other hand, they cannot control the effects of hyperinsulinemia and insulin resistance on PCOS ovulation disorders. Therefore, improving IR and reducing the level of insulin is the key to treatment success. Metformin (Met) is a hot research drug for the treatment of PCOS recently; it has widely used in reducing insulin resistance, weight, menstrual irregularity, and hyperandrogenism.^[11]^ However, the ovulation rate of Met is only 29% to 50%^[12]^ and should take-up at least 6 months to establish a regular menstrual cycle,^[13]^ which has not been as effective as the patient's expectations. Traditional ovulation induction agents combined with Met are only suitable for patients preparing for pregnancy. For patients who currently have no pregnancy requirements, long-term use of ovulation induction agents may increase the risks of endometrial cancer and ovarian cancer.^[14]^ Therefore, it is vital for PCOS patients with IR to find new treatments to improve the efficacy and reduce side effects. Chinese herb medicine, which is an essential complementary therapy, has attracted many interests.

Ethanol extract of *S miltiorrhiza* (Danshen) are a class of bioactive molecules, that is tanshinone. Which is commonly used in China, has been prescribed empirically for the treatment of insulin resistance, hyperandrogenism, acne.^[15–17]^ Various studies have assessed the effect of tanshinone on PCOS. Studies demonstrated that tanshinone could alleviate irregular menstrual, infertility, lipid metabolism disorder, glucose metabolism disorder, etc.^[16,18,19]^ Tanshinone shows an improvement in blood rheology and coagulation of ovaries and uterus,^[20]^ thereby providing an excellent internal environment for follicular growth and development, and power for ovulation. Tanshinone effects on inhibiting carcinogenesis (genes/viruses), can regulate endometrial carcinogenesis and cell proliferation.^[21]^

Through the search of all literature to present, it is found that no systematic review reported the effect of tanshinone on women with PCOS. Here, we will assess the efficacy, as well as the safety of tanshinone for the treating PCOS in women.

## 2 Methods

### 2.1 Registration

This protocol has been registered in the International platform of registered systematic review and meta-analysis protocols (INPLASY) (INPLASY ID: 202050007). We have designed the study as per the maxims of preferred reporting items for systematic reviews and meta-analysis protocols.^[22]^

### 2.2 Eligibility criteria

#### 2.2.1 Study type

This study includes randomized controlled trials (RCTs) of tanshinone or an intergration of tanshinone with other conventional pharmacotherapy to treat PCOS. Non-RCTs will be excluded.

#### 2.2.2 Study object

All eligible patients were defined as diagnosed with PCOS. There will be no limitations on age, origin, and nationality.

#### 2.2.3 Intervention type

We will enroll articles that the intervention arm received tanshinone. All eligible subjects in the control arm will include any type of intervention.

#### 2.2.4 Outcomes

Overall response rate, fasting serum glucose, sex hormone level, fasting insulin, total cholesterol, triglycerides, low-density lipoprotein, high density lipoprotein, ovulation rate, as well as adverse effects.

### 2.3 Search strategy

Two authors will independently search the following electronic repositories: PubMed, Cochrane Library, Web of Science, China National Knowledge Infrastructure (CNKI), CINAHL, as well as EMBASE, to the present. Also, we will retrieve unpublished trials on ClinicalTrials.gov. The search words of the key words will be (“Tanshinone”) and (“polycystic ovary syndrome” or”PCOS”).

### 2.4 Data collection

#### 2.4.1 Study selection

**Figure 1:**
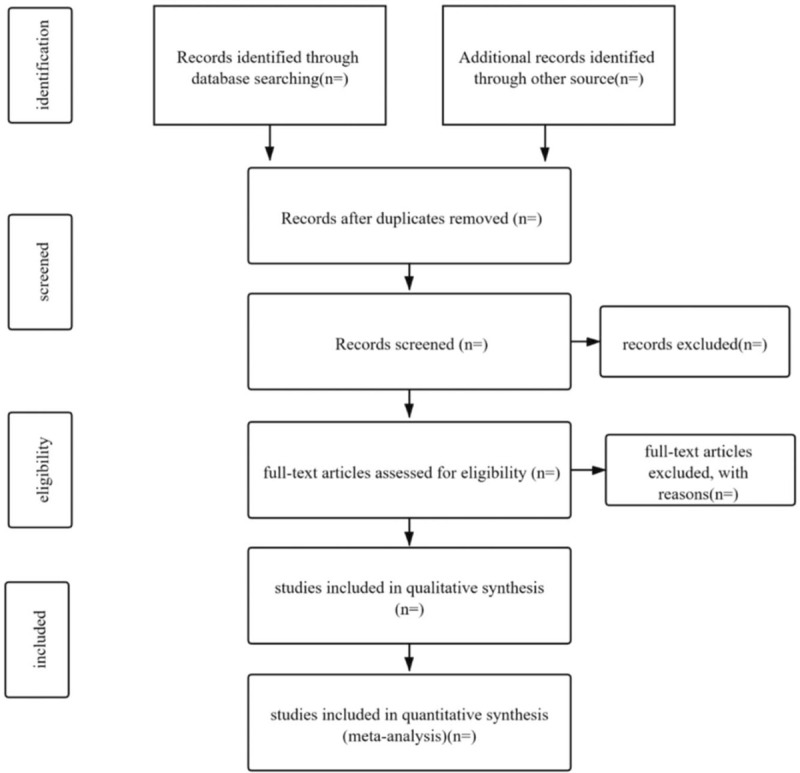
Flow diagram of study selection.

Two independent reviewers shall review all articles (either in English or Chinese) that will be relevant, then through title, as well as abstract scanning select the eligible ones. The complete texts of the studies will be extracted and the studies not meeting the eligibility criteria eliminated. Thereafter, the complete text of all the prospective articles will be read against following the inclusion criteria. In instances of any disagreements between 2 reviewers, a third researcher will be involved to provide solutions to the disagreements via discussions. A preferred reporting items for systematic review and meta-analysis flow chart will be employed to show the article selection criteria (Fig. 1).

#### 2.4.2 Data mining

Two independent researchers will extract detailed data and information from articles, and a third viewer will discuss any disagreement. We will mine the data of necessary information (title, first author, country, year of publication), subjects’ features (sample size, trial design, average age, trial methods, trial setting, the inclusion and exclusion criteria), interventions (doses of tanshinone, type, treatment duration, and follow-up time), comparisons (intervention details, as well as control arms), and outcomes (results, findings, and adverse events).

### 2.5 Date analyses

#### 2.5.1 Measurement of the treatment effect

The Cochrane Review Manager (RevMan 5.3) software will be employed in data analyses. Continuous variable will be estimated by standardized mean difference (MD), as well as 95% confidence intervals (CIs). Moreover, the effect size of dichotomous variables will be estimated by the risk ratio, as well as 95% CIs. The I^2^ statistic will be utilized to indicate statistical diversity. When I^2^ < 50%, we will using a fixed-effect model, otherwise a random-effect model will be employed.

#### 2.5.2 Missing data

If the data were not provided or insufficient, we will contact the authors and for an attempt to get detailed information. When the data are unavailable, we only analyze available data.

#### 2.5.3 Inspection of heterogeneity

We will inspect study heterogeneity by I^2^ statistic, as well as the Chi-squared test, and when *P* < 1 or I^2^ statistic >50%, the studies considered to have significant heterogeneity. If significant heterogeneity is detected, we shall conduct a subgroup analysis.

#### 2.5.4 Inspection of risk of bias

If more than 10 studies are included, we will generate Funnel plots, as well as Egger regression text to analyze publication bias.

#### 2.5.5 Subgroups analysis

We will conduct the subgroup analysis to determine considerable heterogeneity as per study information, patient's features, type of inventing measures, and outcome indicators.

#### 2.5.6 Sensitivity evaluation

The sensitivity inspection will be conducted to inspect the robustness of the study. We will eliminate low qualities articles one by one to inspect the reliability of this meta-analysis’ results.

#### 2.5.7 Quality of evidence

Two authors will evaluate the quality of evidence in systematic review by utilizing the grading of recommendations assessment development and evaluation. The result of the assessment will be divided into high, moderate, low, or very low.

#### 2.5.8 Ethics and dissemination

The systematic reviews with no need for ethics approval because of not involved in individual data.

## 3 Discussion

PCOS is a frequent endocrine disorder in women, it is the principal cause of infertility and amenorrhea.^[23]^ Due to its high recurrence rate, poor prognosis and serious complications, more works on the research of PCOS are needed. In recent years, studies have documented the IR prevalence in PCOS to be higher, reaching 50% to 80%,^[24]^ and it is believed that IR is a direct cause of anovulation. Restoring natural and regular ovulatory menstruation is critical for PCOS treatment, but there is still no perfect treatment. Various ovulation-inducing agents are used for PCOS therapy, but they all have certain deficiencies and side effects. Therefore, traditional Chinese medicine, as the main component of complementary treatments, has attracted attention as a place for the treatment of PCOS.

Among them, tanshinone is a common Chinese patent medicine widely used for PCOS patients who are hyperandrogenism and IR. It is known to affect activating blood and dissolving stasis. There has some studies description of the safety and efficacy of tanshinone. However, systematic review or meta-analysis involving RCT studies designed as tanshinone therapy, is not reported. This study will provide research evidence on the safety, as well as the efficacy of tanshinone in the treatment of PCOS.

## Author contributions

YYJ designed the search strategy and drafted the protocol, and revised by LHL and NXR; XY and PX independently worked on study selection, quality inspection, data mining; YYJ worked on data synthesis; NXR resolved any divergences.

**Investigation:** Xia Peng.

**Methodology:** Yue Xia.

**Resources:** Honglin liu, Yue Xia.

**Supervision:** Yijiao Yang, Jiani Xie.

**Writing – original draft:** Yijiao Yang.

**Writing – review & editing:** Xiaorong Ni.
